# Patients’ Information Needs Related to a Monitoring Implant for Heart Failure: Co-designed Study Based on Affect Stories

**DOI:** 10.2196/38096

**Published:** 2023-01-23

**Authors:** Ambre Davat, Fabienne Martin-Juchat

**Affiliations:** 1 TIMC - Translational Innovation in Medicine and Complexity / Recherche Translationnelle en Médecine et Complexité - UMR 5525 Université Grenoble Alpes La Tronche France; 2 GRESEC - Groupe de Recherche Sur les Enjeux de la Communication Université Grenoble Alpes Échirolles France

**Keywords:** co-design, affect stories, mixed methods study, heart failure, medical implantable device, mobile health, mHealth, remote monitoring, quantified self, telehealth

## Abstract

**Background:**

RealWorld4Clinic is a European consortium that is currently developing an implantable monitoring device for acute heart failure prevention.

**Objective:**

This study aimed to identify the main issues and information needs related to this new cardiac implant from the patients’ perspective.

**Methods:**

A total of 3 patient collaborators were recruited to help us design the study. During 4 remotely held meetings (each lasting for 2 hours), we defined the main questions and hypotheses together. Next, 26 additional interviews were conducted remotely to test these hypotheses. During both phases, we used affect stories, which are life narratives focusing on affect and the relationship between patients and the care ecosystem, to highlight the main social issues that should be addressed by the research according to the patients.

**Results:**

Context of diagnosis, age, and severity of illness strongly influence patient experience. However, these variables do not seem to influence the choice regarding being implanted, which relies mostly on the individual patient’s trust in their physicians. It seems that the major cause of anxiety for the patient is not the implant but the disease itself, although some people may initially be concerned over the idea of *becoming a cyborg*. Remote monitoring of cardiac implants should draw on existing remote disease management programs focusing on a long-term relationship between the patient and their medical team.

**Conclusions:**

Co-design with affect stories is a useful method for quickly identifying the main social issues related to information about a new health technology.

## Introduction

### Background

Heart failure is a serious chronic condition and the leading cause of hospitalization in Europe for people aged >65 years. Because of the aging population, heart failure is considered a major threat to health care systems [1]. Several research projects have therefore been started in recent years aiming at improving heart failure care by using information and communication technologies [2]. In France alone, 6 telemedicine projects were undergoing development in 2018 [3-5], when the first national telemedicine program (ETAPES) was set up to develop and fund real-life remote monitoring [6].

The main issue regarding heart failure management is the prevention of cardiac decompensation, a sudden and life-threatening aggravation of the symptoms that is responsible for frequent hospitalizations of patients with this complication. For now, the detection of cardiac decompensation is mostly based on symptoms reported by the patients, notably weight gain. Physiologic signals recorded by implantable devices would allow earlier detection, resulting in lower rates of hospitalization [7].

### Objectives

RealWorld4Clinic is a research consortium supported by European Institute of Innovation and Technology (EIT) Health that aims to develop MyHeartSentinel, an implantable connected device that could diagnose acute decompensated heart failure 30 days in advance, based on daily recordings of cardiorespiratory data [8]. A unique feature of RealWorld4Clinic is that it involves several researchers in humanities and social sciences, including the Ethics & AI Chair of the Multidisciplinary Institute in Artificial Intelligence of Grenoble Alpes University in Grenoble, France. The objective is to address the ethical, legal, and social issues raised by this new connected medical device early in the innovation process. This approach is inspired by works on ethical health technology assessment [9-11], with an emphasis on patient and public participation [12].

In this paper, we present research that aimed to identify patients’ information needs concerning MyHeartSentinel. For this purpose, we need to better understand patients’ perspectives on heart failure, implants, and remote monitoring.

## Methods

### Overview

Our study is divided into 2 parts. First, we co-designed the main research questions and hypotheses with a small team of patients who were interested not only in following the project but also in collaborating with researchers. Second, we strengthened these hypotheses via a qualitative study involving a wider panel of patients.

In both parts of the study we used affect stories, which are life narratives focusing on affect and relationships. Affect is a significant but long-overlooked part of human experience that is now receiving growing interest [13,14], notably in the design field [15]. By *affect*, we mean any affective phenomena, including feelings, moods, emotions, and attitudes [16]. These affective phenomena are central to social interactions and meaning-making processes [17]. Taking them into consideration is therefore very useful to analyze what matters to patients, what difficulties they face, and how to co-design with them [18].

### Part 1: Co-design of the Main Research Questions and Hypotheses

To recruit our patient collaborators, we contacted RESIC38. RESIC38 is a health network dedicated to heart failure based at the Grenoble Alpes University Hospital. It is in charge of organizing patient pathways and maintaining a therapeutic patient education (TPE) program. It regularly organizes individual or group sessions on various topics, such as “My daily medications,” “Traveling comfortably,” and “Sexuality with a chronic condition.” TPE is an approach in the field of chronic condition management that promotes multidisciplinary and patient-centered care [19]. Since 2009, the French National Authority for Health (Haute Autorité de la Santé) has accredited TPE programs that follow its guidelines. The role of TPE is not only to inform patients but also to help them to adapt medical instructions to their daily lives. TPE therefore promotes patient empowerment and a paradigm shift in the relationship between patient and health care professional [20,21].

The director of RESIC38 conveyed our request to 3 active members of the network—3 men aged 56, 73, and 76 years—whom he considered capable of helping us in our research project. Starting in March 2021, several remote meetings were organized approximately once a month between the authors of this paper and these 3 patients. In the first session, each patient told us his story about heart failure. The patients were asked to expand on the various affective phenomena and social relationships they had experienced during their patient pathway and care pathway. This first session, which was combined with a literature search, allowed us to propose 4 main research questions and a set of hypotheses, which were refined during the second session (Multimedia Appendix 1). In the third session, we proposed a methodology to test these hypotheses, which consisted of collecting evidence from different sources: interviews with patients and health care professionals, literature searches, patient associations’ websites, and health forums. We also discussed the best ways to recruit new interviewees, patients, and health care professionals. At the fourth meeting, we presented and discussed the results of the first interviews. Each session was recorded, and we listened to the recordings to write the minutes, which were then sent to all participants.

### Part 2: Validation of the Co-designed Hypotheses via Qualitative Interviews

Meanwhile, interviews were undertaken to test the co-designed hypotheses. These consisted of nondirected affect stories, completed with some questions. Our interview guide is presented in Multimedia Appendix 2.

Of the 26 interviewees, 19 (73%) were recruited from 2 health networks dedicated to heart failure: RESIC38 (n=8, 42%) and the cardiac unit of Hôpital Privé Le Bois in Lille, France (n=11, 58%). Both provide individualized and multidisciplinary follow-ups with their patients, including drug treatment optimization and patient education, but only RESIC38 organizes group education sessions as part of an official TPE program. Of the remaining 7 participants, 4 (57%) were members of patient associations, and 3 (43%) were contacted having been identified via their relevant posts on social networks.

The sample characteristics are summarized in Table 1. Of the 26 participants, 16 (62%) were men, and 10 (38%) were women. The average age was 65 (SD 17; range 21-89) years. Most (21/26, 81%) of the patients had already been implanted with a medical device with remote monitoring capabilities, and some (12/18, 67%) were part of a remote follow-up program using connected objects. Most (21/26, 81%) of the patients were living with heart failure, and 12% (3/26) had experienced it before receiving a heart transplant. The (2/26, 8%) exceptions were patients with suspected cardiac disease who had contacted us after receiving the recruitment advertisement by mistake. One of these interviews was particularly interesting for us because an implantable loop recorder was mentioned. This is a diagnostic tool used to record cardiac data, but it cannot deliver electrical impulses to regulate the heartbeat. It is close to the implant MyHeartSentinel in terms of form and implantation procedure.

**Table 1:** Participant characteristics (N=26).

Characteristics		Values, n (%)
**Gender**		
	Man	16 (62)
	Woman	10 (38)
**Age (years)**		
	<50	4 (15)
	50-65	8 (31)
	>65	14 (54)
**Professional activity**		
	Active	5 (19)
	Unemployed	6 (23)
	Retired	15 (58)
**Socioprofessional group (before retirement or disablement)**		
	Farmer	0 (0)
	Artisan, merchant, business executive	3 (12)
	Upper managerial or intellectual occupation	7 (27)
	Intermediate profession	8 (31)
	Employee	5 (19)
	Blue-collar worker	3 (12)
**Time (years) since first cardiac follow-up**		
	<2	5 (19)
	2-10	7 (27)
	10-30	11 (42)
	>30	3 (12)
**Implants**		
	Defibrillator	12 (46)
	Pacemaker	5 (19)
	Heart transplant	3 (12)
	Implantable loop recorder	1 (4)
	None	5 (19)

There was no prior relationship with any participant. An informed consent document was sent to each participant and re-explained at the beginning of each interview. All of the participants agreed to be recorded. They were interviewed remotely for approximately 1 hour, either by videoconferencing or by telephone. On 3 occasions the participant’s partner was also present and intervened during the interview.

Each interview was replayed once and summarized by the interviewer (AD). The portions referring to affective experience or relationships were transcribed verbatim.

Information that could be used to identify the patients was either generalized (eg, city names were replaced by brief sociodemographic information) or anonymized (eg, in the case of physicians’ names).

### Ethical Considerations

According to French legislation, our study did not require ethics approval because our aim was not to develop biological or medical knowledge. However, we sought and received approval from the multidisciplinary ethics committee of Grenoble Alpes University (CERGA-Avis-2021-24), which checked the compliance of our interviews with the European General Data Protection Regulation.

## Results

### Overview

Our analysis is mostly deductive, based on the research questions and hypotheses codefined with the patient collaborators. In the following paragraphs, we present our results according to this reading grid (the hypotheses are presented in Multimedia Appendix 1). We have selected some representative quotes, which have been translated from French into English.

### Question 1: Are There Patient Profiles for Which the Implant Is (or Is Not) Appropriate? In Particular, Is It Necessary for the Patient to Accept the Disease Before Entering a Monitoring Program?

#### Overview

The experience of living with heart failure seemed to vary significantly from one interviewee to another. To better understand these different perspectives, we analyzed similarities and dissimilarities among the affect stories. We identified 3 key factors that strongly influence the patient experience: context of diagnosis, age, and illness severity.

#### Context of Diagnosis

Some people became patients living with heart failure overnight after an emergency hospitalization for myocardial infarction or acute pulmonary edema. Thus, they discovered intensive care and the world of cardiology for the first time. On their return home, they had to learn to live with a new chronic condition, as observed by a patient:

I had never been sick. I mean severely ill. This was my greatest wealth...When they told me that I had to be anesthetized, I was terrified. I had never been anesthetized in my life!...All my life, I had never taken my blood pressure. So uh...I learned to do all that...I have the greatest difficulty with accepting myself as “being sick.”

P17, woman aged 80 years hospitalized a few months previously for myocardial infarction

Others *slide* progressively into heart failure after years of cardiac follow-up. Their perception of the disease and the relationship with the medical community may therefore be quite different from those patients who take ill suddenly. A patient stated as follows:

I have seen people at the RESIC, who were on top form and suddenly...a shock. Before that, they could run, etc...I, however, have never been able to run the 100 meters, you see? So it didn’t change my life.

P9, man aged 86 years diagnosed with a heart defect in childhood

Between these 2 extreme examples, there is a great diversity of trajectories. In particular, many (8/26, 31%) of the patients knew that they had a family history of cardiac issues, which nevertheless did not prevent them from being startled by their first hospitalization. This family history is a source of concern, but it is also a means by which they can picture themselves in the future, as explained by a patient:

When the cardiologist told me that the results of my ultrasound were not good, I collapsed, because all the images of my ill father were brought back and I thought: “This time, it’s my turn.”...When I understood I had the same pathology (we compared the medical records), I knew that I would not escape a heart transplant. And thus, I had the time to prepare mentally, while my father didn’t. He was so afraid that he gave up. He gave up and died very quickly.

P7, man aged 45 years and heart transplant recipient

#### Age

Although heart failure is very frequent among those aged >65 years, it can occur at any age [22]. We observed significant differences between the experiences of younger patients and those of older patients.

Professional activity is a major issue for younger patients. They often need accommodation at work or professional retraining, especially if they have a physical job. Sometimes they are not able to work any longer and instead have to survive on disability allowance. Another concern is parenthood because pregnancy is discouraged for patients with a cardiac condition, and taking care of children is more difficult because of the physical limitations and uncertainty associated with the disease. For these patients, heart failure is an invisible disability that is hard to reconcile with social conventions. Sometimes, they are reluctant to use the assistance to which they are entitled, such as reserved parking places, because of what people might say. Therefore, they must learn to deal both with the need not to look ill and the need to conserve their limited energy.

Fatigue and breathlessness are more easily accepted among older adult patients or even downplayed. As in the case of younger patients, they feel the loss of their physical capabilities, but they do not attribute it only to heart failure. They often experience several pathologies: not only cardiovascular issues (such as hypertension) or diabetes but also respiratory illness, sleep apnea, visual or auditory impairment, osteoporosis, loss of balance, dementia, and so on. As a consequence, they tend to take many medications, which increases the risks of unwanted side effects and unobservance. Optimizing their treatment requires many trade-offs. To take just 1 typical case: a participant (a man aged 83 years) explained that he was advised by his cardiologist to stop diuretics to preserve renal function, but very soon he had to resume his usual medication because of water retention.

Heart failure is not always the patient’s main concern, especially if it is at an early stage; for example, a patient aged 73 years, who had been successfully treated for cancer 10 years previously, reported that when receiving his blood test results, he was more worried about tumor markers than about heart failure markers. Sometimes, the patients are also caregivers for their partner, which causes them considerable anxiety and affects their finances if their partner needs to be moved to a nursing home.

#### Illness Severity

Heart failure severity is usually evaluated according to the New York Heart Association functional classification system [23]. This system consists of 4 classes based on the symptoms reported by patients and how these symptoms affect their daily lives by limiting their physical activities. A patient in class I does not show any symptom of cardiac impairment, whereas a patient in class IV is unable to undertake any physical activity (including walking) and may experience fatigue, palpitation, dyspnea, or angina pain even at rest. Disease management aims at reducing these symptoms and slowing down disease progression. If the disease is advanced and resistant to treatment, heart transplantation is the last resort, but it is a rare and dangerous operation, reserved for patients with the greater benefit-risk ratio.

It is worth noting that our study participants did not mention their New York Heart Association class. However, they frequently talked about their left ventricular ejection fraction (LVEF) value and how it had evolved since their diagnosis. LVEF describes the efficacy of the heart in pumping blood. The LVEF value may rise with disease management or fall in cases of aggravation.

The aforementioned factors influence the difficulties faced by patients and thus their individual level of illness acceptance, that is, their psychological adaptation to the illness [24]. It is clear from the interviews that this acceptance takes time and that it is not always possible for the patients to accept their illness. Indeed, some of them cannot bear the thought of losing their physical capabilities; for example, an interviewee told us that before his transplantation he had tried to keep cycling as though he was not ill at the risk of aggravating his heart condition. Another explained that he was working part time as a consultant. Should he stop working, he stated, it would be “the end of everything.” However, neither patient was reluctant to participate in management of his disease or to test new treatments; on the contrary, they were keen to do so to improve their physical condition. This suggests that illness acceptance is not essential for a patient to accept a monitoring implant. On the contrary, it could be seen at first glance as a way to escape the illness by delegating self-monitoring to the device. This would not necessarily be a problem, provided that remote monitoring works and that the patients have access to a TPE program when they are ready to be more involved in the management of their disease. In this case, the implant would be akin to *Ariadne’s thread*, connecting the patients to their health care teams and maybe even to their peers.

Indeed, most of the interviewees thought that interaction with other patients was very important. These interactions could either be supervised by a medical team as part of a TPE program or initiated by the patients themselves. We used a thematic analysis to understand what they were seeking in these interactions (Multimedia Appendix 3).

### Question 2: What Are the Determining Factors That Would Lead Someone to Accept or Reject a Monitoring Implant?

At the time of the interviews, most (18/21, 86%) of the patients who had experienced the implantation of a cardiac prosthesis (including the 3 persons who wore a defibrillator before receiving a heart transplant) seemed to consider it as just another step on their patient pathway. They mentioned it briefly and sometimes did not even do so until they were questioned about their follow-up. When they expressed their feelings, they thought first about what this implant meant regarding their health condition:

[About her defibrillator] That’s what has marked my transition to serious heart problems.

P4, woman aged 50 years and heart transplant recipient

[After my first hospitalization], it was a second shock, more violent, because I thought: “It’s not a pacemaker, because apparently my heart is beating, but it might race.

P7, man aged 45 years and heart transplant recipient

[About her pacemaker] It is all the better for me because it means that they think that my health is good enough to benefit from it.

P23, woman aged 83 years

These patients consented quickly to the implantation as part of their treatment, trusting their cardiologist’s advice. Most (15/21, 71%) of them had been implanted with a defibrillator and mentioned that the device gave them a sense of safety. For those who had had to wear a cardiac LifeVest for months, the implantation was even a relief because they no longer had to live with a wearable defibrillator day and night, as explained by a patient:

This LifeVest...It was horrible. It weighs two and a half kilos, and you always need to carry it. When I was walking I carried it...It is far better to insert the defibrillator as I have it now. Because even at night I should keep it and sleep with it. It was not a panacea.

P21, man aged 75 years

Only 14% (3/21) of the patients delayed their implantation for as long as possible: P10, P13 and P14 (see next section). They emphasized the importance of receiving moral support to overcome their concerns:

The only person who helped me, it was when I got my defibrillator: the Social Security and the physicians gave me a psychologist for three sessions...My cardiologist had told me a while ago that I should get a defibrillator. He told me that for three or four years: defibrillator, defibrillator...He is a super guy, so I said: “Well, we’ll see...” Finally, he gave me this defibrillator. Hum...it went well, but I took a moral blow anyway. I suddenly became much grayer.

P10, man aged 79 years

At 50 years old I was not very happy about having a foreign object in my body...I was wondering if my physical abilities would degrade in relation to this implant. It’s very important to have moral support. I did not have it. I insisted for three years on not being implanted and finally I accepted, and I am happy to have done it because I had a heart attack one year later.

P13, man aged 55 years

This confirms our first hypothesis: the determining factor in the acceptance or refusal of an implant is the trusting relationship with a health care team. Oudshoorn [25] has even suggested that patients do not really have a choice because these implants are the present standard of care.

To go further, we analyzed the conditions required for this trusting relationship, based on both the positive and negative experiences reported by the participants. The emerging themes and subthemes are summarized in Multimedia Appendix 4, along with some quotations. Many of these can be linked to TPE, as suggested in our second and third hypotheses (Multimedia Appendix 1).

### Question 3: What Are the Main Sources of Anxiety Related to the Implantation of a Monitoring Device?

It is already known that many cardiac implant wearers face anxiety or depression [26,27]. However, as previously mentioned, our study participants did not talk very much about their implants; instead, their feelings of anxiety seemed to be linked more to the severity and unpredictability of their illness. In other words, they are afraid of dying:

I didn’t dare to do sport too much on my own anymore because I was really scared of an accident, of my heart racing, because I had been told it was the main concern.

P7, man aged 45 years and heart transplant recipient

The longest thing was...I was back home and whenever I fell asleep at night I thought: “Will I wake up tomorrow?” Because it was a close one. The odds were against me and I was lucky to survive...So, afterwards, you stay a long time with this idea: “Will I wake up tomorrow?” While thinking: “Anyway, what can I do about it?”

P25, man aged 73 years

I am old, but not in my head. And that’s what I struggle to accept, because I love to tinker and things like that...Morally, it is like a blow because I am always wondering: “How will it evolve? Can I plan something in three months...six months...?” I don’t have the answer.

P21, man aged 75 years

The sources of anxiety listed in our hypotheses were mentioned but as inconveniences rather than as deterrents, which is consistent with the results of prior studies based on the Florida Patient Acceptance Survey [28-30]. Our interpretation is that the patients’ concerns are mostly linked to their illness and its consequences for their daily lives and mortality, rather than to the implant itself. However, our interviewees have either never experienced defibrillator shocks or experienced them only on very rare and appropriate occasions. The situation is certainly very different for patients experiencing multiple shocks [31,32].

Whether these results are easily transposable to monitoring implants is unclear. Implanting a monitoring implant under the skin is safer than implanting a pacemaker or a defibrillator, the leads for which can be a source of medical complications [33,34]. However, there is greater public awareness about pacemakers and defibrillators than about monitoring devices. Moreover, pacemakers and defibrillators actively contribute to the health of their wearer, whereas a cardiac monitor may seem excessive if it is presented only as a diagnostic tool; for example, a short paper in 2012 reported that among 1093 patients with kidney failure screened for a pilot study, 372 were found to be suitable, and only 8 were accepted to receive an implantable cardiac monitor [35]. Later studies were more successful, probably thanks to the miniaturization of the device [36]. Some research has even explored how the implantable loop recorder is perceived by patients [37,38].

To better understand patients’ motives for rejecting implants, another theme should be explored: the “foreign body*”* or *cyborg* theme. Indeed, some patients are concerned not only about the impact of the implant on their lives but also about its mere presence inside their body. Among our 21 study participants who experienced implanted medical devices, 2 (10%) had asked their cardiologists whether their implant could eventually be removed if they got better, or after their death. Another noted that his friends make fun of him by calling him a robot and that his defibrillator’s wires are visible on his x-ray images. In extreme cases, patients may perceive the implantation to be a dangerous operation and a threat to their human identity. This was the reaction of the interviewee P14 who wore an implantable loop recorder. This person was a retired nurse aged 70 years. Because she experienced transient ischemic attacks (ministrokes), she was sent by her referring physician to a cardiac rhythmologist to find the cause of these events. But the consultation went wrong: the patient was flabbergasted at the sight of the device and strongly disagreed with having this “foreign body” inside her. She blamed the cardiac rhythmologist for running out of patience when she started asking questions, as if everything were already decided. After her referring physician insisted, she finally agreed to meet another cardiac rhythmologist and ultimately consented to the implantation.

### Question 4: What Is the Impact of a Monitoring Device on the Patient Pathway?

Different experiences of remote follow-up were reported during the interviews.

The patients (n=11) recruited via the Hôpital Privé Le Bois were (or had previously been) part of a remote monitoring program. Each day they measured their weight and blood pressure and filled out a symptom survey via a set of connected objects provided by the hospital. Even those who had limited experience of IT had no difficulty using these devices. As part of a home return assistance program (PRADO, the French acronym for Posthospitalization Home Return Assistance Program), some (2/11, 18%) of them also received visits from a nurse during the 2- to 6-month period after their last hospitalization. These participants were genuinely surprised at the quality of their follow-up. When their symptoms increase or if they do not use the devices for a couple of days, they immediately receive a call from a nurse who checks up on their situation daily. These calls are seen as proof of the existence and effectiveness of the monitoring program:

Every morning, I take my blood pressure, I weigh myself, and I send all that to the monitoring center. I don’t know where it is, I don’t know who...who takes my stuff. But it works quite well, because on occasion I left for a weekend or a couple of days and I didn’t bring all these things which were a little heavy in the suitcase with me. And they didn’t miss! The nurse called me saying: “Mister X, are you feeling well?” So it’s well followed up.

P18, man aged 75 years

Every morning, around 11 AM, 11:30 AM, they receive all the results. If necessary, they call me. If it’s not necessary, well, they don’t call. And if there is nothing at all during one or two weeks, they call me anyway. It’s more to catch up. They said: “Don’t worry, there is nothing, it’s just to check on how you feel whether everything alright, whether you’re not anxious.” This phone call is really...a comforting touch.

P24, man aged 61 years

The patients thus feel reassured by this program. Of the 11 patients, 3 (27%) mentioned that they were more motivated to watch their weight because someone was watching over them. They stated that they can even feel empowered by this approach:

In the clinic, I was in a bit of a strange psychological state. Because every morning, I faced myself in the mirror as a patient. And when I received visits from these ladies, I was like, I am with you right now: more like making a contribution to an action, on something. You see? Well, it was a great help anyways...It’s wonderful because we feel surrounded and supported. You see, it’s like a kind of...partnership. I live it as a kind of partnership. Everyone has a place, of course, I am not a cardiologist or a specialized nurse. But it’s a...a dialogue actually. It allows people to be an actor of their health, we can say it like that.

P17, woman aged 80 years

Of the 11 patients in this program, 10 (91%) also had a pacemaker or a defibrillator whose proper functioning was monitored by the same nurses. Opinion on remote monitoring seemed to be more divided among the other patients with implants outside of this program (n=12). Of these 12 patients, 2 (17%) had actually been contacted by the hospital because of a malfunction of their implants and estimated that they were well monitored, but 5 (42%) expressed doubts that the monitoring was really effective because they had never received a call when they experienced arrhythmia or even a shock from their defibrillator. They stated that they regretted having to call the hospital themselves to obtain information:

I think it could be a useful tool if it was monitored. Sometimes I had alerts, but no phone call. Whereas I was told, “As soon as we’ll see an episode, we’ll inform you.” I needed that to be reassured somehow, but what seemed odd to me was that when there were alerts, I was the one who had to seek information, instead of information coming to me thanks to the monitoring people.

P7, man aged 45 years and heart transplant recipient

As has already been shown by Skov et al [39], many patients are not satisfied with a “No news is good news” approach. They need to directly and repeatedly experience that there is actually “someone at the end of the wire” to trust the remote monitoring [40]. Moreover, in the absence of a program dedicated to remote monitoring, the follow-up of cardiac implants seems to be less diligent and coordinated because it is provided by nurses who have other duties to attend to. A Swedish research study on remote monitoring showed that nurses struggle to manage alerts from multiple interfaces (one for each manufacturer) and that the time required to do so was not always acknowledged by their managers [41].

Another difference between the 2 patient groups could be seen in their access to their health data. The patients from the Hôpital Privé Le Bois have access to their measures and their history, which became a conversation topic with the health care professionals and a learning opportunity, as noted by the interviewees:

Suddenly my blood pressure rose from 9/8 to 12/8. I was wondering: “What does it mean?” It was strange...I talked about it with the nurse and we reviewed the previous records together and it was alright...I realized that maybe I should be more careful about what I do between my breakfast and my measurement.

P17, woman aged 80 years

I saw the nephrologist yesterday and he said I was taking my blood pressure too fast and I should be more relaxed. Surely my blood pressure was lower this morning. But I may have made a mistake because I took my medications first...I think I should take my blood pressure before.

P23, woman aged 83 years

By contrast, patients who wear a cardiac implant have no free and immediate access to their own data, the analysis of which is performed by specialized cardiologists whom they meet only once or twice a year. Even if the patients write down dates and times when they feel an abnormal sensation, these notes do not match the physician’s observations. Conversely, abnormal recordings are very difficult to link with their experience, as noted by a participant:

My defibrillator records tachycardia episodes. Surprisingly, it does not correspond to a particular fatigue or overactivity...Sometimes I feel bad. I can’t explain what it is, but I don’t feel well. So I note it and when I go to the control visit, I ask, “What happened at this time?” “Nothing. Everything was fine.” But two months later, bang! There is a burst.

P3, man aged 76 years

Eventually, the patients need to use additional measuring instruments such as smartwatches to monitor their heart rate when they exercise and avoid experiencing a shock from their defibrillator. In the case of an audible alert from their device, they may not recognize it immediately or know what to do, as stated by a participant:

One day, it’s rare but it happens, the wire to the heart broke. You see? Of course, I didn’t know it, but I felt bad. It was the first time I was hearing a small ring inside me. I told my wife: “Do you hear a ring? It’s like a phone ringing.” And she said: “No, no. There is no phone ringing.” But it was inside, you see. It’s weird when you’re not used to it...I went to the shower, I grabbed the shower head, or something else. I passed out and I woke up sitting in the shower. That’s when I understood...While my daughter was driving me to the hospital, I collapsed maybe five or six times because I didn’t know that each time I raised my left arm, the contact was lost.

P8, man aged 63 years

It seems that TPE is completely overlooked in the design of current cardiac implants, which focus solely on sustaining heart function. The development of a new monitoring device could provide an opportunity for patient empowerment. However, Lomborg et al [32] have shown that access to self-tracking data has ambivalent effects. It may be a tool to promote self-care, but it can also be a cause of frustration and distress when the patients are not able to associate the data with their sensations and emotions. This issue could be even more acute with implantable medical devices [42], whose measures are supposed to be accurate and reliable. It is also expected that, in some cases, the data will have very sensitive implications for the patient. If their illness is worsening, they should be informed first by a physician, not by an application.

## Discussion

In this final section, we will discuss not only the implications of this study on the development of an information medium related to MyHeartSentinel but also the transformation in patient monitoring brought about by the development of such health implants.

### Pros and Cons of an Implantable Monitoring Device

MyHeartSentinel is an implantable device, which has both advantages and drawbacks. Among its advantages, it allows trustworthy and automatic measurements. Compared with the CardioMEMS implant [43,44], this subcutaneous implant is less invasive, and once the gateway is installed, data transmission will not require any commitment (a priori) on the part of the patient. It is already known that adherence to self-care is an important issue in the field of chronic conditions [45], including heart failure [46-48]. Therefore, it seems interesting to use low-invasive implants to deliver medication [49] or, in our case, to monitor patients living with heart failure. Ideally, a team of health care professionals should be dedicated to remote monitoring to quickly respond to any alert and to support their patients in disease management.

In terms of drawbacks, we can expect patients to be reluctant to agree to the implantation. Diverse reasons have already been mentioned in our paper: fear of surgery, threat to personal privacy, transformations in daily life and the relationship with the health care team, and fear of becoming a cyborg. The success of this particular implant will depend on whether SentinHealth (the medical technology start-up developing MyHeartSentinel) will be able to convince cardiologists that its device is relevant, both in terms of medical outcomes and organizational routines.

### Patient Empowerment and Co-design as a Condition to Effective Disease Management

Close follow-up of medical data is certainly useful, but it will not be sufficient to improve heart failure management. Our interviews as well as reviews on remote monitoring showed that the success of remote monitoring is highly context dependent [2-5,50-52]. The important thing is not just which remote monitoring system should be used but also how it should be used and by whom. It seems essential to co-design not only with patients but also with every stakeholder as much as possible, and the process should include the technical device itself as well as the clinical routines and information supports [53-57].

Another challenge, identified by Greenhalgh et al [2], is the possibility of “tinkering” with remote monitoring to adjust to each particular situation, which may seem contradictory given the values of standardization and quantitative performance that are generally associated with automated systems. In our study, we collected a great variety of narratives, which we analyzed through the prism of 3 key variables: context of diagnosis, age, and illness severity. Analysis through other lenses such as gender, social class, psychology, culture, or isolation would certainly lead to interesting conclusions [58-61]. In the face of this complexity, it seems difficult to define relevant patient profiles. Rather than a one-size-fits-all approach focused on one of these profiles, we think that it is better to consider a modular and customizable follow-up program; for instance, patients would decide with their physicians which information and services they should have access to. This could be renegotiated over time, according to patients’ readiness to participate in the remote monitoring program. In this case, clinical evaluation should not be limited only to hospital admission and survival rates but should also include patient-centered outcomes, self-defined by each patient [52].

### Limitations

Although diversified by gender, sociocultural background, location, age, and time since diagnosis, our sample is not representative of the entire population of patients living with heart failure. This is a methodological problem observed in many studies requiring patient involvement [55,56,62]. The 3 patient collaborators as well as most of the interviewed patients are highly educated (17/26, 65%), able to speak with ease (23/26, 88%), and are willing to share their experiences. Issues related to health care access or literacy may therefore be underestimated.

Moreover, our recruitment method did not allow us to access patients who are uninformed or in denial about their disease, which is certainly a big issue in heart failure. According to the ICPS2 survey administered in 2018 to nearly 800 patients hospitalized for acute heart failure at 40 centers, 1 in 3 patients was not able to name the disease [63].

It should be noted that MyHeartSentinel was still in development at the time of publication of this paper. We based our study on interviews with patients who had had experiences of heart failure, implants, and remote monitoring, but none of them wore the new implant yet. This study was therefore undertaken to understand what kind of conditions would be required for patients to accept the new implant, and the results will need to be confirmed with future patients.

### Conclusions

We have presented research aimed at identifying the issues and information needs related to an implantable monitoring device for patients with heart failure. After co-designing the hypotheses of the study with a small team of patient collaborators with a methodology based on affect stories, we tested the hypotheses via 26 additional interviews. Most of the initial hypotheses were validated, and some were rephrased or completed by our observations. None was discarded. This confirms that co-design with affect stories is an effective method for quickly identifying social issues related to a new health technology.

We found that the monitoring implant should be conceived primarily as a mediation instrument, rather than as a quantified self tool, that facilitates illness acceptance and communication between patients and health care professionals. The results of this study will be used to design the prototypes of an information module in collaboration with user experience designers at SentinHealth.

## Appendix

Multimedia Appendix 1 Research questions and hypotheses co-designed with the patients.

Multimedia Appendix 2 Interview guide.

Multimedia Appendix 3 Motives for interacting with other patients.

Multimedia Appendix 4 Health care professionals' qualities.

## Abbreviations

EIT: European Institute of Innovation and Technology
LVEF: left ventricular ejection fraction
PRADO: Posthospitalization Home Return Assistance Program
TPE: therapeutic patient education
