# Supplementary material for: Patients’ Information Needs Related to a Monitoring Implant for Heart Failure: Co-designed Study Based on Affect Stories
Source: JMIR Hum Factors. 2023 Jan 23;10:e38096. doi: 10.2196/38096 (PMC9947817; doi:10.2196/38096)
Supplement: Multimedia Appendix 1 [file humanfactors_v10i1e38096_app1.docx]

**The four research questions and hypotheses co-designed with the patients**

| 1) Are there patient profiles for which the implant is (or is not) appropriate? In particular, is it necessary for the patient to “accept” the disease before entering a monitoring program?   - Rather than identifying patient profiles, we need to model the temporality of illness acceptance. - A monitoring implant can be an additional means by which to secure patients and help them to initiate a process of illness adaptation / acceptance.   - A patient network would be a huge help in answering patient concerns, in supporting their illness adaptation, and in training them with the numerical tool.   2) What are the determining factors which would lead someone to accept/reject a monitoring implant?   - The determining factor is the trusting relationship with a healthcare team.   - This relationship relies on the caregiver training in the field of therapeutic patient education.     - Therapeutic patient education is essential in illness acceptance.   3) What are the main sources of anxiety related to the implantation of a monitoring device?   - The risks associated with surgery - The risks connected to personal data (cybercrime, denial of insurance…) - The consequences for the quality of everyday life (implant visibility, body sensations, constraints…) - The consequences for the relationship with the healthcare team (loss of social link).   4) What is the impact of a monitoring device on the patient pathway?   - The remote follow-up of heart failure through implants requires the emergence of new stakeholders.   - These may be healthcare professionals in charge of monitoring health data, or technical advisors in charge of resolving computer issues. - The implant must be associated with monitoring services (e.g., a mobile application), which would allow the patient:   - to access their health data   - to better understand their illness via a quantification of their symptoms   - to have a special relationship with their healthcare team. |
| --- |
