# Supplementary material for: Patients’ Information Needs Related to a Monitoring Implant for Heart Failure: Co-designed Study Based on Affect Stories
Source: JMIR Hum Factors. 2023 Jan 23;10:e38096. doi: 10.2196/38096 (PMC9947817; doi:10.2196/38096)
Supplement: Multimedia Appendix 2 [file humanfactors_v10i1e38096_app2.docx]

**Interview guide**

*This guide contains the main topics we wish to address during the affect story, and some questions to relaunch the interviewee. The aim is not to follow it to the letter as a semi-structured interview guide, but to allow the participant to express themselves freely and to invite them to elaborate their narrative. In the case of relaunch, efforts will be made to avoid leading questions, by reusing the words of the participant.*

1. **Context of diagnosis**

What was your life situation when your first symptoms appeared? (Age, profession…)
How was the diagnosis made? (When? Who? Where? ...)

If the interviewee wears a cardiac implant:
 Who presented you with this implant?
 Do you remember if you asked questions at this time? What were your questions?
 How did you take this decision?

1. **Follow-up**

How are you followed-up for your heart failure? (Who? When? Where?)
Do you manage to make medical appointments?
Who do you trust most among your healthcare professionals?
Are you followed-up in telecardiology? What do you think of that?

1. **Daily life**

Today, how is your daily life with heart failure?
Have you gave up some activities or hobbies? Why?
Have you started new activities?
Do you use connected objects to manage your health?

1. **Social and professional relationships**

Has heart failure had an impact on your social relationships? (familial, friendly, or professional)

Why did you join this patient association / health network?

1. **Information materials**

Did you search for information on heart failure? How?
What do you think is the best information source?
