# Supplementary material for: Patients’ Information Needs Related to a Monitoring Implant for Heart Failure: Co-designed Study Based on Affect Stories
Source: JMIR Hum Factors. 2023 Jan 23;10:e38096. doi: 10.2196/38096 (PMC9947817; doi:10.2196/38096)
Supplement: Multimedia Appendix 3 [file humanfactors_v10i1e38096_app3.docx]

**Motives for interacting with other patients**

**Theme: Social needs**

| Subthemes | Quotes |
| --- | --- |
| Fighting loneliness | *I created a Facebook group thinking: “Maybe there is a way to find some folks who live with the same thing as me.”*  [P16, man aged 33 years] |
|  | *I needed to not feel alone. Because the cardiologists said: “You know, you’re one case in…” So you feel a little like an alien with their figures. Seeing other people who live with it like you… I had this need to feel that my reaction of not being in “national grief” was legitimate.* [P6, woman aged 35 years] |
| Finding a purpose | *Then I created an association. It was a motor, because I had the feeling of being useful despite the wait (for transplantation).* [P16, man aged 33 years] |
| Comparing with others | *I attended two meetings at the RESIC, one with my wife. When I saw the people around me… They were very, very tired. Much younger than me and very tired. So it confirmed my… my lifestyle.* [P10, man aged 79 years] |
|  | *Now, every time I hear of someone with a heart issue, immediately I try to listen, to see what they have more than my husband, what they have less, whether it’s easier for them to live with.* [P26, wife of a patient aged 64 years] |

**Theme: Access to information**

| Subthemes | Quotes |
| --- | --- |
| Sharing experiences | *At the RESIC, I attended all the sessions which were proposed to me, sometimes twice. It really helped me in learning what I should do or not do, listen, etc. Thus it was very valuable to me.* [P7, man aged 45 years and heart transplant recipient] |
|  | *In the session about traveling, a patient told that he used to take a picture of his prescription with his phone. That is good advice!* [P2, man aged 73 years] |
| Balancing the doctor’s viewpoint | *I speak a lot on Instagram with people, girls who were transplanted. There is precisely a girl who were transplanted in the same hospital as me and who is almost the same age as me. Thus, I know a lot more thanks to these people because their opinions are completely different from those of the cardiologists, or the anesthetists, etc. So I am well informed. On the other hand, I don’t want to know too much, so… I limit myself to the minimum: What I need to know.* [P5, woman aged 21 years] |
|  | *The clinicians are not the only ones who need to change their posture, but also the patients. We are conditioned because the physicians know all. They have all the power. There is a joke about the cardiologists: “What is the difference between God and a cardiologist?” You know it. Sometimes this image is in the patient’s mind, but it’s not my way of dealing with it. It’s my heart, it’s my body. I am the one who lives with it, not the physician.* [P13, man aged 55 years] |
