# Supplementary material for: Patients’ Information Needs Related to a Monitoring Implant for Heart Failure: Co-designed Study Based on Affect Stories
Source: JMIR Hum Factors. 2023 Jan 23;10:e38096. doi: 10.2196/38096 (PMC9947817; doi:10.2196/38096)
Supplement: Multimedia Appendix 4 [file humanfactors_v10i1e38096_app4.docx]

**Healthcare professionals’ qualities**

**Theme: Material organization**

| Subthemes | Quotes |
| --- | --- |
| Spatial and temporal reachability | *Doctor X no longer takes patients in the clinic since COVID. He is at his office in [town]. But soon, he will move to [another location] so it will be closer. There is the parking also. He knows it is difficult for me to go there. He liked to see us every three months, but it’s been six months since we last met. Now, he is following up with his device, so I think if there was really a problem, the doctor would call me.* [P26, wife of a patient aged 64 years] |
|  | *I am lucky to have a cardiologist who is efficient and who knows that I don’t call her for peanuts. So when I call her, she gives me an appointment as soon as possible.* [P18, man aged 75 years] |
| Records management | I am dissatisfied with a lot of things at hospital X, but I stay there because it is where all my files are. There are tons and tons… I can’t picture myself changing hospital. It is not sustainable. [P4, woman aged 50 years and heart transplant recipient] |

**Theme: Expertise**

| Subthemes | Quotes |
| --- | --- |
| Reputation | *I consulted a professor who was the number 1 of the hospital, who was well-known.* [P10, man aged 79 years] |
|  | *At the time of my recruitment, they asked: “Who follows you?” “Doctor X.” “Then we have nothing to say.” You see. I was told that in [two different cities] so I trusted in him.* [P9, man aged 86 years] |
|  | *They have a very bad reputation. Thus, I did not want at all to be followed-up in [my local hospital].* [P22, man aged 59 years] |
| Competence | *Usually I prefer to talk to my cardiologist rather than to my referring physician. Because… It’s not that he knows nothing, but it’s not his domain. He’s more general.* [P5, woman aged 21 years] |
|  | *Since I was taken in charge in [city of the University Hospital], I have stayed there. My town is a small one, you know. They are not specialists about the heart. While the University Hospital, sure, it’s an important center.* [P8, man aged 63 years] |

**Theme: Good communication**

| Subthemes | Quotes |
| --- | --- |
| Clarity | *The cardiologist is simple, so I am like him. We are able to ask questions and to understand, because sometimes the physicians… they answer but with their words*. [P26, wife of a patient aged 64 years] |
|  | *In this hospital, everyone was lovely. About heart failure, they always explained it to me very well. They repeated: “If you have other questions, ask them.”* [P5, woman aged 21 years] |
|  | *[About the pre-transplantation consultation meeting] This is very well done. There is nothing to say. The one who says that he was not aware of the smallest detail before the operation is a liar. They use simple, understandable terms. It is not the… medical semantics which can be sometimes used to… when you are here and you don’t have a clue about what is said.* [P22, man aged 59 years] |
| Transparency | *I can’t get along with this cardiologist, because it’s someone who gives no info. I can’t bear it… Sometimes I do my blood test in town in order to have the results, because it’s more complicated to get them from the hospital.* [P4, woman aged 50 years and heart transplant recipient] |
|  | *The first cardiologist I saw in the hospital when I arrived in the emergency room gave me no information. I found out I had heart failure on the report I read at home. I found that… a little outrageous.* [P15, woman aged 59 years] |
|  | *I received a mail after the second consultation meeting. It confirmed what was said… And there, a paragraph was added saying that they could put me a… an artificial heart awaiting transplantation… That had not been mentioned, and there is no way I would walk around with a device next to me… No, no, no.* [P22, man aged 59 years] |
|  | *When you don’t have as much information as you wish, you don’t know what will fall on you.* [P11, woman aged 77 years] |

**Theme: Human relationship**

| Subthemes | Quotes |
| --- | --- |
| Acquaintance | *I entirely trust Doctor X because she is the one who operated on me and who follows me on a regular basis.* [P8, man aged 63 years] |
|  | *They said: “We have to think about transplantation.“ But it was not my usual cardiologist who told me, it was a cardiologist I didn’t even know. A professor. Usually I am on good terms with every caregiver, cardiologist, etc. But the professors, I can’t.* [P5, woman aged 21 years] |
| Care | *In the emergency room, I didn’t feel listened to. […] You’re just a number among many others. […] My cardiologist, he is very attentive. I can feel it is not assembly-line methods.* [P6, woman aged 35 years] |
|  | *My town cardiologist… I will not blame him, but I didn’t find him very concerned by the thing, so I quickly withdrew towards Doctor X at the day hospital. [P7, man aged 45 years and heart transplant recipient]* |
|  | *When I was operated on, the professor came to see me before the operation to see what state I was in. He had been retired for four years at this time. On a human level, it touched me a lot*. [P9, man aged 86 years] |

**Theme: Partnership**

| Subthemes | Quotes |
| --- | --- |
| Taking care of the patient’s viewpoint | *Normally, people like me cannot have children, because… actually being pregnant increases cardiac output by 30%. So it’s very dangerous. I said I wanted children, and because my cardio was very exceptional he prescribed me a battery of tests… and I had two children.* [P4, woman aged 50 years and heart transplant recipient] |
|  | *They give you a picture of transplantation which is far from idyllic. Anyway, I took the decision to be transplanted. […] I was very, very surprised when I saw Doctor X arriving in my room saying: “I have a good news for you: Your medical condition has improved and you will be removed from the transplantation list.” I said: “You’re kidding, is this good news?!”* [P22, man aged 59 years] |
| Team-work with other healthcare professionals | *Every six months I go to the cardiologist. Last time, I brought my follow-ups diary. Well… He didn’t write anything. While my referring physician checks every time I see him. And the nurse conscientiously fills the pages which are reserved for her. The cardiologist, even if I brought it, he wrote nothing at all. If he does not play the game…* [P23, woman aged 83 years] |
|  | *When I was in the hospital for the second time, they called the nephrologist. And when the cardiologist sees me, he sends the report to the nephrologist and vice versa. And the cardiologist who follows me, who takes care of my pacemaker, he works too with them… There is a big team of cardiologists in the clinic. For example, during my second hospitalization, he handed me over another cardiologist who specializes in heart failure, in order to review my therapeutics etc. Well… It works with mutual understanding.* [P25, man aged 73 years] |
|  | *I keep seeing the cardiologist who implanted my pacemaker. […] Last time, she found that I should do something more about the heart: the burn I told you about.* *I said: “Alright, but I wish I could have talked to my regular cardiologist first”. I tried, but there was a problem and I could not get an appointment in time. Thus the operation was done without his endorsement. […] He was upset we did that without his advice. And next time I went to his office, he checked on me of course, but then he said: “Mister X, I will no longer heal you.”* [P12, man aged 89 years] |
